# Control of Residues of Antimicrobial Substances by Screening Methods in Raw Milk Based on Participation in Proficiency Tests in Poland, 2017–2021

**DOI:** 10.3390/foods11172635

**Published:** 2022-08-30

**Authors:** Magdalena Łuszczyńska, Marlena Gołaś-Prądzyńska, Jolanta Grażyna Rola

**Affiliations:** Department of Hygiene of Food of Animal Origin, National Veterinary Research Institute, 24100 Puławy, Poland

**Keywords:** proficiency testing, antimicrobial residue, raw milk, screening methods

## Abstract

Proficiency testing (PT) is an important tool for evaluation of the competences of laboratories which test milk for residues of antimicrobial substances. It also warrants the reliability of the obtained test results, which is important to the clients of a laboratory. In 2017–2021, the Polish National Reference Laboratory organized 10 rounds of proficiency testing on raw milk samples according to the ISO/IEC 17043 standard. The milk samples were fortified with selected antimicrobial substances. All participating laboratories used commercial receptor and/or microbiological diagnostic kits in the proficiency tests. The results obtained by the laboratories were compared to assigned results and were defined as compliant or non-compliant. In total, 7533 results were obtained, and 104 (1.4%) were assessed as non-compliant. The percentage of laboratories which obtained a positive result in proficiency tests ranged by testing round from 81.8% to 100%. Based on proficiency testing results, it can be concluded that laboratories carry out tests correctly. The proven proficiency by a laboratory increases the confidence of its clients in its service of antimicrobial residue monitoring in milk.

## 1. Introduction

The problem of the proper use of antibiotics in animal husbandry and treatment still seems to be relevant and to be one which obligates authorities to monitor harmful substances (chemical and biological) in food of animal origin permanently and requires continuous improvement of methods in order to ensure food safety. One of the safety aspects is the determination of limits that are safe for humans and the constant monitoring of food for compliance with them. The relevant limits were specified in Commission Regulation (EC) No 37/2010 [1]. As a result of the imposition of limits, laboratories are required to ensure the appropriate quality of monitoring tests, which has an impact on the safety of food of animal origin. Uncontrolled residues of antimicrobial substances in food products could be a threat to human health, leading to allergic reactions, hypersensitivity, aplastic anemia, the disruption of the balance of the gastrointestinal microflora by inhibition of the growth of lactic acid bacteria, and the generation of resistant intestinal bacteria. Antimicrobial substances may also disrupt the natural microflora of milk and dairy products, and prevent the proper production of dairy products by inhibiting the growth of starter cultures, which may bring financial losses for the dairy industry [2,3,4]. One of the elements of monitoring of residues of antimicrobial substances in milk is the national program of tests for the presence of prohibited substances, chemical and biological residues and medicinal products in animals and food of animal origin. An important element of that national program is the verification of the competence of the laboratories in it, achieved by their participation in proficiency testing (PT) [5]. The PT organizer should meet the accreditation requirements specified in the PN-EN ISO/IEC 17043 standard, which formally confirm its competence to perform tasks in the field of conformity assessment [6]. Proficiency testing gives the opportunity to confirm the competence of a given laboratory to carry out tests by comparing it with other participants and establishes objective measures to evaluate and demonstrate the reliability of the obtained test results [5]. It also affords a means of detecting possible errors and taking appropriate corrective actions. Positive results in regular participation in proficiency tests confirm the correctness of the applied procedures and test methods, and thus increase client confidence in laboratories and control authorities [6].

The aim of this study is to analyze the results of the proficiency tests in the detection of residues of antimicrobial substances by screening methods in raw milk which were organized by the Polish National Reference Laboratory (NRL) in 2017–2021.

## 2. Materials and Methods

### 2.1. Organization of Proficiency Tests

The organizer of the proficiency tests was the Department of Hygiene of Food of Animal Origin of the National Veterinary Research Institute in Puławy, which acts as the National Reference Laboratory (NRL) for residues of antimicrobial substances from group B1 [7]. An NRL is obliged to organize proficiency tests for official laboratories and use the obtained results [8,9]. The obligation of regular participation in proficiency tests is imposed on the Veterinary Inspectorate Laboratories (of the Department of Veterinary Hygiene) as well as laboratories performing tests, of which the results are used for the purposes of official surveillance [9,10]. Obtaining a positive result in PT is one of the requirements that must be met in order to be entered in the Chief Veterinary Officer’s (CVO) registry of raw milk testing laboratories as well as one of the requirements needed for a laboratory to be approved to perform specific tests [11]. Obtaining unsatisfactory results in two consecutive rounds of proficiency testing or failing to participate regularly in ring tests causes the laboratory to be removed from the register or its approval withdrawn. Detailed provisions regulating these issues are contained in arts 25a and 25e of the Veterinary Inspection Act [9]. In addition to official laboratories and raw milk testing laboratories registered with the CVO, other laboratories conducting routine food tests, including private laboratories, may also participate in tests of proficiency in detection of residues of antimicrobial substances. For laboratories of the Veterinary Inspectorate, participation in comparative tests is free, while other entities pay the fee specified in the terms and conditions for participation.

### 2.2. Participants

The participants of proficiency tests were Laboratories for Official Control (Veterinary Inspectorate Laboratories and CVO approved Laboratories), laboratories from the CVO registry, and other diary laboratories conducting routine milk tests.

**Figure 1:**
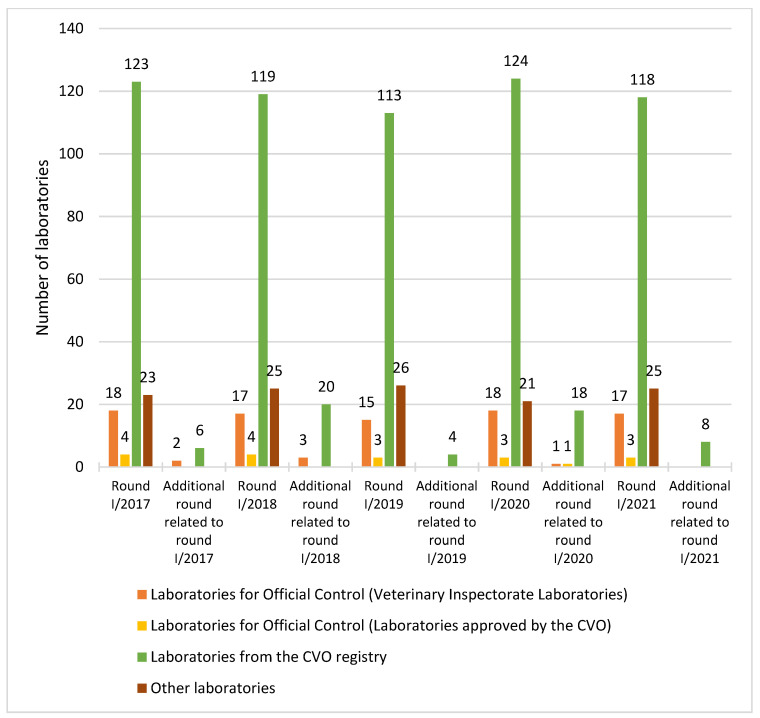
Number of laboratories participating in proficiency testing rounds using raw milk samples in 2017–2021. CVO—Chief Veterinary Officer.

There were 157 to 168 participants in the proficiency testing rounds, not including participants in additional rounds. The largest group of PT participants was laboratories from the CVO registry, which accounted for 72% to 75% of all participants in individual rounds. The number of laboratories participating in each round of proficiency testing is shown in Figure 1.

### 2.3. Development and Implementation of PT

Information on proficiency tests for detection of residues of antibacterial substances is available at www.piwet.pulawy.pl (accessed on 1 July 2022) in the “Oferta” under “Badania Biegłości” (proficiency testing) and Zakład Higieny Żywności Pochodzenia (Department of Hygiene of Food of Animal Origin). The inhibitory substances of which residues come within the scope of tests are detailed, and the terms and conditions of proficiency tests with the schedule of rounds for the calendar year are given. During the year, the National Veterinary Research Institute organizes one round of proficiency testing using raw milk as a matrix. For laboratories from the CVO registry and official laboratories, in case of unsatisfactory proficiency test results, additional rounds are organized in accordance with the legal regulations [9].

The proficiency testing program’s execution consisted of many stages, including composing the terms and conditions of participation, preparing sample handling instructions, preparing test samples and checking their homogeneity and stability, shipping samples to participants, analyzing the submitted results, evaluating them, and drafting a PT report. Application for participation in proficiency tests and reporting of the results was performed through the e-klient electronic system of registration, information, transfer and, analysis of data (www.eklient.piwet.pulawy.pl (accessed on 1 July 2022)); participants received a code for their laboratory, which ensured the anonymity of the laboratory. Participants could use any number of methods for testing, but laboratories from the CVO registry were required to use the declared methods, and official laboratories used accredited methods or methods submitted for accreditation.

According to the PT schedule, a parcel containing random coded samples of milk kept frozen in conditions enabling the maintenance of the appropriate temperature (styrofoam thermo-packaging with cooling inserts) was shipped to the registered participants. Each package included 3 or 6 frozen raw milk samples, cooling inserts, and a temperature logger (this monitored temperature during transport). The samples were kept frozen until shipment. Upon receipt of the samples, the laboratory assessed their suitability for testing and was required to start testing within 24 h. The participant could withdraw from the test if the quality of the samples raised doubts by notifying the organizer immediately.

### 2.4. Preparation of the Materials

The PT was organized entirely by the laboratory, and no subcontracting was permitted. The absence of residues of antibiotics in the “blank” material was checked by three screening methods validated in the laboratory: one microbiological—Delvotest SP-NT (DSM, Delft, The Netherlands) and two receptor-based methods: 4Sensor (Unisensor, Seraing, Belgium) for the detection of beta-lactams, tetracyclines, dihydrostreptomycin/streptomycin and chloramphenicol residues and Charm ROSA MRL BL/TET (Charm Sciences Inc., Lawrence, MA, USA) for the detection of tetracycline and beta-lactam residues. Tested and confirmed antimicrobial-free milk was fortified with selected antimicrobial substances so that the final concentration of the active substance was close to European Union (EU) requirements, maximum residue limit (MRL) or minimum required performance level (MRPL) value and the required detection level of the method according to the requirements of the Customs Union (CU) [1,12]. Appropriate solvents were used to dissolve the substances, and the final concentration was prepared in raw milk, free of antibacterial substances. For each participant, a set of 3 samples was prepared according to the EU requirements and a set of 3 samples according to the CU requirements, from 2019, 1 sample in the set was negative (antibiotic-free sample). The antimicrobial substances used in the proficiency testing are presented in Table 1.

### 2.5. Homogeneity

An analysis of the homogeneity was performed for each material on the day the samples were prepared according to the harmonized protocol for the proficiency testing of analytical laboratories [13]. For each material, the procedure was as follows:

- 10 containers were randomly selected from the prepared containers,
- 2 × 10 aliquots were analyzed on the same day using three laboratory-validated procedures for antibiotic residues: one microbiological—Delvotest SP-NT (DSM, Delft, The Netherlands) and two receptor-based methods: 4Sensor (Unisensor, Seraing, Belgium) for the detection of beta-lactam, tetracyclines, dihydrostreptomycin/streptomycin and chloramphenicol residue and Charm ROSA MRL BL/TET (Charm Sciences Inc., Lawrance, MA, USA) for the detection of tetracycline and beta-lactam residues.

The homogeneity criteria were as follows: the milk material was considered homogeneous when each of the antibiotic residues was fully detected in the sample. After evaluation, the contaminated milk materials were considered homogeneous according to the above criteria. For the milk material free of antibiotic residues material, the criterion was the absence of any antibiotic residues in the 2 × 10 portions. The blank material was considered homogeneous.

### 2.6. Stability

The stability of the milk materials was also evaluated according to the harmonised protocol for the proficiency testing of analytical laboratories [13].

On the day the samples were tested by the participants, 3 containers of each material were randomly selected from the remaining containers stored at <−15 °C. The contents of each of the 3 containers were then homogenized and 2 portions were taken from each for testing; 2 × 3 portions were analyzed on the same day using laboratory-validated procedures for antibiotic residues. The criterion for stability was as follows: the milk material was considered stable if the antibiotic residue was detected in all the test portions. During the analysis period, the dairy materials were considered sufficiently stable. Complete agreement of all obtained results was assumed as a criterion of the homogeneity and stability of samples. When assessing the homogeneity and stability of control samples, the National Reference Laboratory used three methods of detecting residues of antimicrobial substances, which are accredited by the Polish Center for Accreditation and bear certificate AB 485. Raw milk samples were tested using the three methods described above in Section 2.5. 

### 2.7. Screening Methods Used by Proficiency Test Participants

The assay market offers a wide range of different tests based on microbiological and receptor methods, which can be used for antimicrobial residue monitoring in milk and dairy products.

Microbiological methods are based on the principle of inhibiting the growth of the *Geobacillus stearothermophilus* test strain by antimicrobial substances contained in the tested milk. These methods are relatively simple to perform and do not require expensive equipment, but lead to false positive results because of naturally inhibiting substances such as lactoferrins and milk lactoperoxidases. The advantage of microbiological methods is their capacity to detect a wide range of substances in a large number of samples simultaneously. The disadvantage is the inability to identify specific substances (qualitative methods) and the relatively low level of detection of some antibiotics. These methods are characterized by high sensitivity to beta-lactams, but much lower sensitivity to tetracyclines, sulfonamides, macrolides, or chloramphenicol [14]. The presence of antibacterial substances inhibits the growth of the test strain with no discoloration of the agar medium. In the absence of antibacterial substances in the tested milk, the growth of the test strain is not inhibited, causing the acidification of the medium and a color change to yellow. The test result is obtained after 2.5–3 h of incubation at 63–65 °C. Participants in proficiency testing mainly used the following tests: Delvotest SP-NT, Delvotest T and Delvotest BR Brilliant (DSM, Delft, The Netherlands), CowSide II (Charm Sciences Inc., Lawrence, MA, USA), Polutest M and Polutest MS (POLUTEST Barbara Kawiorska, Olsztyn, Poland), BRT Screening test (AiM GmbH, Breisgau, Germany), ECLIPSE 50 (Zeulab, Zaragoza, Spain), and Milchtest MT 288 FP (Packhaus Rockmann GmbH, Sendenhorst, Germany). 

Receptor methods are based on the binding of antimicrobial substances to specific receptors on the paper strip along which milk diffuses. These methods can detect various groups or single antibacterial substances, and their advantage is the short result time which is just minutes. Most often, these tests detect residues of tetracyclines and beta-lactams, but despite the similarities of receptor method tests, they differ in sensitivity to individual substances. Participants in proficiency testing mainly used the following tests: 4Sensor BSCT-KIT060, Twinsensor BT KIT020 and Twinsensor BT KIT034 (Unisensor, Seraing, Belgium), BetaStar S Combo and BetaStar Combo (Neogen Co., Seraing, Belgium), Charm MRL BL/TET, Charm MRL BL, Charm MRL, Charm Chloraphenicol Test, Charm Streptomycin Test, Charm MRL BLRFTET 2 and Charm QUAD Test (Charm Sciences Inc., Lawrence, MA, USA), Delvotest BLF (DSM, Delft, The Netherlands), Bioeasy 2in1 (Shenzhen Bioeasy Biotechnology Co., Shenzhen, China), Delvotest Fast BT (DSM, Delft, The Netherlands), MilkSafe™ 4BTSC/BOX and MilkSafe™ 3BTC/BOX (Shenzhen Bioeasy Biotechnology Co., Shenzhen, China).

### 2.8. Criteria for Evaluating the Results

The criterion for assessing the results obtained by the laboratories participating in PT was their matching of the assigned result, owing to the qualitative nature of the methods used. A result compliant with the assigned value was determined when the laboratory detected the presence of an antibiotic in a positive sample (sample containing antibiotic), or presence of an antibiotic that has not been detected in a negative sample (antibiotic-free sample). A result non-compliant with the assigned value was determined when the laboratory did not detect the presence of an antibiotic in a positive sample (sample containing antibiotic) or detected the presence of an antibiotic in a negative sample (antibiotic-free sample). The results of the proficiency tests of the participants were categorized each time as compliant or non-compliant with regard to the method used and the sensitivity declared by the manufacturer of the test. The evaluation of a laboratory participating in testing, of its proficiency in particular European Union or Custom Union requirements, depends on the judgement of the results achieved by that laboratory. It was assumed that the laboratory merited an unsatisfactory proficiency test result if only one of the submitted results was assessed as non-compliant. Participation in an additional round of proficiency testing is obligatory for Veterinary Inspectorate Laboratories and laboratories from the CVO registry.

## 3. Results

In the analyzed period, 10 rounds of proficiency testing with milk samples were organized, comprising five main rounds of proficiency testing and five additional rounds. 

In each round, participants received samples in accordance with the schedule and which are appropriate to the declared field of assaying. Participants submitted a total of 7533 results, of which 104 were assessed as non-compliant (1.4%). In two rounds, three participants did not submit their results by the deadline. These laboratories were not included in the assessment. All milk-testing laboratories used commercial receptor and/or microbiological diagnostic kits.

The analysis of the results showed that the microbiological methods, yielded 81 non-compliant results, while the receptor methods gave 23 such results (Table 2). Table 3 presents a detailed description of the non-compliant results obtained. The most common among these results (substances not detected—false negative results) were obtained with microbiological methods for samples fortified with gentamicin or substances from the tetracycline group. This mainly concerned the following tests: Delvotest SP-NT, Delvotest T, CowSide II, and Polutest MS. Among the receptor-based methods, false negative results (substance non-detected) were obtained mainly with the Twinsensor BT KIT020 test. These discrepancies concerned the samples fortified with tetracyclines (Table 3). During the analyzed period, 14 (0.19% of all results) of the samples free from antimicrobial substances were non-compliant, i.e., were false-positive results. In four rounds, participants detected antimicrobial substances (tetracyclines and oxacillin) below the detection limit by the manufacturer and these results were classified as compliant on the basis of the documentation provided by the test manufacturers. This mainly concerned the detection of samples containing tetracyclines (93.9% of results) by microbiological tests: Delvotest T, CowSide II, and BRT Screening Test. Oxacillin-fortified samples were detected by participants below the manufacturer’s declared limit of detection using the Charm MRL BL/TET receptor test.

**Figure 2:**
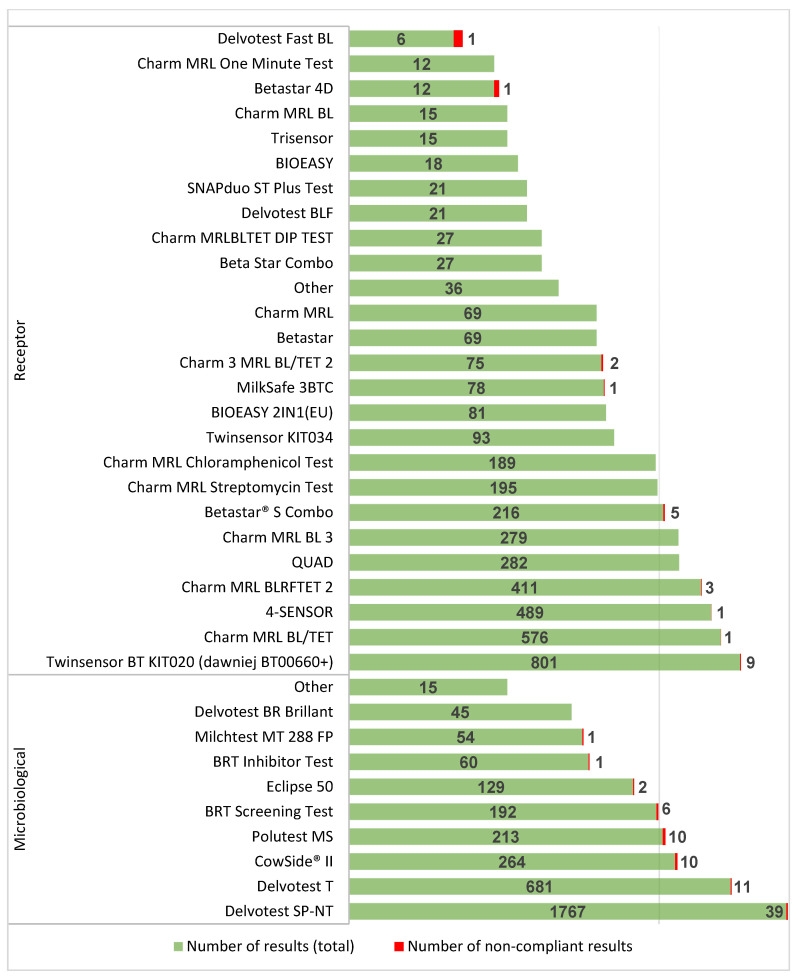
Microbiological and receptor test results by the test product with indications of the number of non-compliant results in 2017–2021.

In the years 2017–2021, participants in proficiency testing used the most common receptor tests. Most results were submitted using the following tests: Twinsensor BT KIT020 (801 results), Charm MRL BL/TET (576 results), 4Sensor (489 results), and Charm MRL BLRFTET 2 (411 results). In the analyzed period, the most frequently used tests based on the microbiological method were: Delvotest SP-NT (1767 results) and Delvotest T (681 results). Non-compliant results were obtained mostly with microbiological methods (Delvotest SP-NT—39, Delvotest T—11, CowSide II—10, and Polutest MS—10), and among the receptor methods, non-compliant results occurred sporadically. Detailed data are presented in Figure 2.

**Figure 3:**
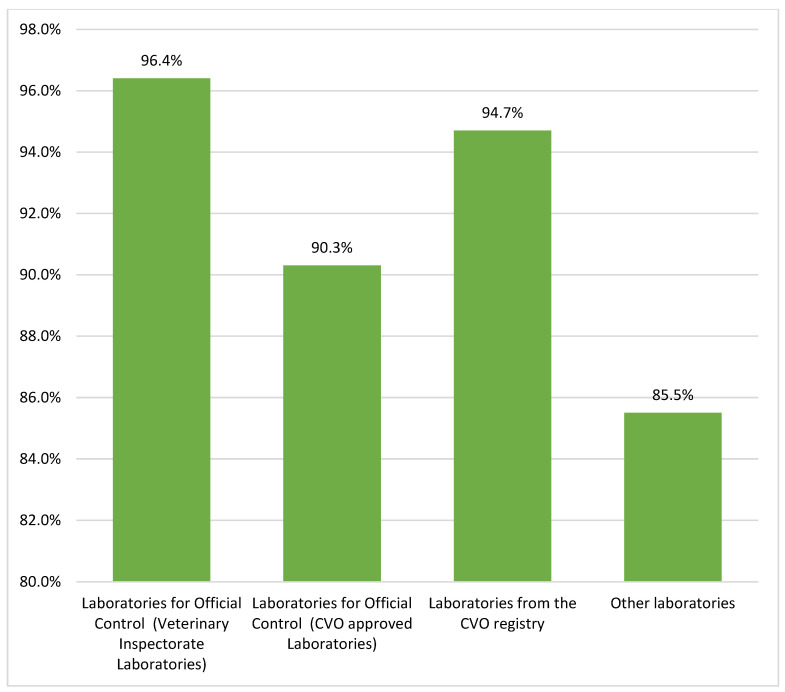
Percentage of laboratories with a positive evaluation in PT from raw milk samples in 2017–2021.

The percentage of laboratories which passed the proficiency tests in particular main rounds ranged from 81.8% to 100% (Table 1). The percentage of laboratories with a positive PT result is presented in Figure 3.

## 4. Conclusions

The organization of proficiency testing is a time-consuming and labor-intensive process, starting with the drawing up of a schedule of proficiency testing and ending with the drafting of a test report [15]. The analysis of the results of proficiency tests in 2017–2021 for residues of antimicrobial substances allowed the NRL as the organizer to objectively assess the work of the participants in comparison to the results obtained by other laboratories. These data indicated the high level of competence of the assessed laboratories. The percentage of official laboratories with a positive assessment was 93.35%. The presented data confirmed that the official laboratories and laboratories of the CVO registry perform the procedures for the detection of antimicrobial residues correctly; the results of the tests routinely performed by dairy laboratories for their own purposes are also satisfactory.

Non-compliant results were obtained quite rarely (1.4%), compared to the results of Różańska et. al. where the percentage of such results was higher at 2.1% [15]. The submitted results indicate one of the basic reasons for non-compliant results to be mistakes made by laboratory staff, e.g., wrong interpretation of the result or incorrect filling in of the scorecard. The main problem with microbiological methods was the interpretation of the results (false positive or false negative results), and with the receptor-based methods, the lack of detection of antimicrobial substances. Compared to the results of PT in 2013–2016, an increase in the number of inconsistent results obtained with microbiological methods and a decrease in those given by receptor-based methods was observed. Additionally, a significant drop in the number of laboratories with an unsatisfactory PT result in additional rounds was observed [15]. It is worth noting that the simultaneous use of microbiological and receptor methods increases the range and sensitivity of detection of specified substances in milk. Obtaining an unsatisfactory result in proficiency testing does not disqualify the laboratory unless it occurs regularly. It facilitates the correction of errors and implementation of corrective actions, enables continuous improvement of the laboratory, and prevents future mistakes.

## Figures and Tables

**Table 1:** Antimicrobial substances used for proficiency testing according to EU and CU requirements in 2017–2021.

Year	Substance (Company)	Concentration (µg/kg)	MRL/MRPL * (µg/kg)
2017	ampicillin (Sigma-Aldrich, St. Louis, MI, USA)	5	4
	tylosin (Sigma-Aldrich, St. Louis, MI, USA)	100	50
	tetracycline (Sigma-Aldrich, St. Louis, MI, USA)	10 **	100
	chloramphenicol (Sigma-Aldrich, St. Louis, MI, USA)	0.3	0.3 *
	dihydrostreptomycin (Sigma-Aldrich, St. Louis, MI, USA))	200	200
	Tetracycline (Sigma-Aldrich, St. Louis, MI, USA)	100	100
	oxytetracycline (Sigma-Aldrich, St. Louis, MI, USA)	10 **	100
2018	gentamicin (Sigma-Aldrich, St. Louis, MI, USA))	100	100
	tetracycline (Sigma-Aldrich, St. Louis, MI, USA)	30	100
	chloramphenicol (Sigma-Aldrich, St. Louis, MI, USA)	0.3	0.3 *
	streptomycin (Sigma-Aldrich, St. Louis, MI, USA)	200	200
	tetracycline (Sigma-Aldrich, St. Louis, MI, USA)	100	100
	neomycin (Sigma-Aldrich, St. Louis, MI, USA)	500	1500
2019	oxacillin (Sigma-Aldrich, St. Louis, MI, USA)	30	30
	chlortetracycline (Sigma-Aldrich, St. Louis, MI, USA)	100	100
	chlortetracycline (Sigma-Aldrich, St. Louis, MI, USA)	10 **	100
	chloramphenicol (Sigma-Aldrich, St. Louis, MI, USA)	0.3	0.3 *
2020	tetracycline (Sigma-Aldrich, St. Louis, MI, USA)	100	100
	cephapirin (Sigma-Aldrich, St. Louis, MI, USA)	60	60
	dihydrostreptomycin (Sigma-Aldrich, St. Louis, MI, USA)	200	200
	tetracycline (Sigma-Aldrich, St. Louis, MI, USA)	10 **	100
2021	cefalonium (Sigma-Aldrich, St. Louis, MI, USA)	20	20
	chlortetracycline (Sigma-Aldrich, St. Louis, MI, USA)	100	100
	chlortetracycline (Sigma-Aldrich, St. Louis, MI, USA)	10 **	100
	penicillin G (Sigma-Aldrich, St. Louis, MI, USA)	4	4

* Minimum Required Performance Level for chloramphenicol. ** Concentrations according Custom Union requirements.

**Table 2:** Summary of proficiency test results in 2017–2021.

Proficiency Test (PT) Round	Number of Participants	Number of Results (Total)	Number of Non-Compliant Results			Number of Laboratories with an Unsatisfactory PT Result	Percentage of Laboratories with a Positive PT Evaluation
			Total (%)	Microbiological Methods	Receptor-Based Methods		
Round I/2017	168	1548 ^(1)^	18 (1.2%)	8	10	12	98.3%
Additional round related to round I/2017	8	51	0	0	0	0	100%
Round I/2018	165	1470 ^(2)^	32 (2.2%)	31	1	30	81.8%
Additional round related to round I/2018	23	108	3 (2.8%)	2	1	1	95.6%
Round I/2019	157	1359 ^(3)^	8 (0.6%)	6	2	7	95.5%
Additional round related to round I/2019	4	18	0	0	0	0	100%
Round I/2020	166	1416 ^(4,5)^	32 (2.3%)	28	4	25	85%
Additional round related to round I/2020	20	117	0	0	0	0	100%
Round I/2021	163	1416 ^(6)^	11 (0.8%)	6	5	10	93.9%
Additional round related to round I/2021	8	30	0	0	0	0	100%
Total		7533	104 (1.4%)	81	23		

Comments: ^(1)^ Two laboratories did not send the results by the required deadline. ^(2)^ Nineteen laboratories detected antimicrobial substances below the detection limit declared by the manufacturer with the microbiological method and three laboratories with the receptor-based method. ^(3)^ Fifty laboratories detected antibacterial substances below the detection limit declared by the manufacturer with the microbiological method and 39 laboratories did so with the receptor-based method. ^(4)^ One laboratory did not submit results by the required deadline. ^(5)^ Eleven laboratories detected antimicrobial substances below the detection limit declared by the manufacturer with the microbiological method and three laboratories did so with the receptor-based method. ^(6)^ Nine laboratories detected antimicrobial substances below the detection limit declared by the manufacturer.

**Table 3:** Summary of non-compliant results for the tested samples.

Year		2017				2018				2019					2020					2021			Total
Antimicrobial Substance		Ampicillin 5 µg/kg	Tylosin 100 µg/kg	Tetracycline 10 µg/kg	Blank	Tetracycline 100 µg/kg	Gentamicin 100 µg/kg	Blank	Streptomicin 200 µg/kg	Oxacillin 30 µg/kg	Chlortetracycline 100 µg/kg	Chlortetracycline 10 µg/kg	Chloramphenicol 0,3 µg/kg	Blank	Tetracycline 10 µg/kg	Tetracycline 100 µg/kg	Cefapirin 60 µg/kg	Dihydrostreptomicin 200 µg/kg	Blank	Chlortetracycline 100 µg/kg	Cefalonium 20 µg/kg	Blank	
Microbiological	Delvotest SP-NT				1	1	24	1	1						2	7		1				1	39
	BRT Screening test							1						1						4			6
	BRT Inhibitor Test		1																				1
	Delvotest T	1	1		1	1	3			1						3							11
	CowSide II															8	1		1				10
	ECLIPSE 50										1					1							2
	Polutest MS		1		2			1			2					2	1		1				10
	Milchtest MT 288 FP																			1			1
Receptor	Charm MRL BL/TET					1																	1
	Charm MRL BLRFTET2	1		1									1										3
	Charm 3MRLBLTET2	1										1											2
	Delvotest Fast BL									1													1
	Twinsensor BT KIT020	1	1	2		1										3			1				9
	4Sensor	1																					1
	MilkSafe 3BTC																			1			1
	BetaStar S Combo	2																		1	1	1	5
	BetaStar 4D																					1	1
**Total**		7	4	3	4	4	27	3	1	2	3	1	1	1	2	24	2	1	3	7	1	3	

## Data Availability

Data is contained within the article.
